# Clinical Determinants and Bone Metabolic Correlates of 24-h Urinary PGE2 and PGEM Excretion in Chinese Adults: A Multicenter Cross-Sectional Study

**DOI:** 10.3390/biomedicines14071547

**Published:** 2026-07-10

**Authors:** Qi Lu, Li Shen, Yang Xu, Zhenlin Zhang

**Affiliations:** 1 Shanghai Clinical Research Center of Bone Disease, Department of Osteoporosis and Bone Diseases, Sixth People’s Hospital Affiliated to Shanghai Jiao Tong University School of Medicine, Shanghai 200025, China; luqi1997@sjtu.edu.cn (Q.L.); shenli11@sjtu.edu.cn (L.S.); 2 Clinical Research Center, Sixth People’s Hospital Affiliated to Shanghai Jiao Tong University School of Medicine, Shanghai 200025, China

**Keywords:** 24 h urinary prostaglandin E_2_ excretion, 24 h urinary PGE metabolite excretion, clinical determinants, bone metabolism markers, multicenter cross-sectional study

## Abstract

**Background**: Prostaglandin E_2_ (PGE_2_) is a key lipid mediator involved in inflammation and bone homeostasis. Its systemic production is reliably reflected by 24 h urinary excretion of PGE_2_ (U-PGE_2_) and its major metabolite (U-PGEM). However, the physiological association between systemic PGE_2_ production, calcium-phosphorus homeostasis and bone turnover markers remains unclear. This study aims to elucidate these relationships in a general Chinese adult population. **Methods**: In this multicenter, cross-sectional study, 737 Chinese adults underwent standardized 24 h urine collection. Multivariable linear regression was used to assess independent associations with bone metabolism markers. Restricted cubic spline models were further employed to examine nonlinear relationships. **Results**: The median 24 h U-PGE_2_ and U-PGEM excretion levels were 133.87 and 246.76 pg/mmol creatinine, respectively, with no significant sex differences (both *p* > 0.05). Multivariable regression analyses revealed that both 24 h U-PGE_2_ and U-PGEM were independently and positively associated with advancing age. Notably, both 24 h U-PGE_2_ and U-PGEM maintained a significant inverse association with serum calcium (Overall *p* < 0.05). Restricted cubic spline analyses further demonstrated a significant non-linear association between both 24 h U-PGE_2_ and U-PGEM and total procollagen type 1 N-propeptide (P1NP; both Overall *p* < 0.05). This relationship was characterized by a steep decline in U-PGE_2_ and U-PGEM excretion at lower P1NP concentrations, which subsequently plateaued at higher concentrations (Overall *p* < 0.05). Additionally, U-PGEM exhibited a significant inverse linear association with intact parathyroid hormone (PTH; Overall *p* < 0.05). **Conclusions**: This study provided valuable insights into the clinical determinants of 24 h U-PGE_2_ and U-PGEM in Chinese adults and their independent associations with calcium-phosphorus homeostasis and bone turnover markers.

## 1. Introduction

Prostaglandin E_2_ (PGE_2_), a potent lipid mediator derived from arachidonic acid, plays multifaceted roles in human physiology, including the regulation of inflammation, angiogenesis, tissue repair, immune surveillance and bone homeostasis, through G protein-coupled receptor subtypes (EP1-EP4) [1,2,3]. The biological effects of PGE_2_ are largely determined by receptor types on target cells and the local PGE_2_ concentration [4,5]. For instance, binding to the EP1 receptor, PGE_2_ mediates pain perception and smooth muscle contraction, while binding to the EP3 receptor, it plays an important role in fever and vasoconstriction [6,7,8]. The PGE_2_-EP2 axis is involved in age-related inflammation and cognitive decline, while the PGE_2_-EP4 axis regulates bone formation and promotes muscle stem cell repair [3,9,10]. Moreover, PGE_2_ exhibits concentration-dependent characteristics, ranging from homeostatic maintenance at low levels, to immunomodulation at moderate levels, and eventually to tissue repair or pathological promotion at high levels [11,12,13].

In addition to physiological roles, abnormally elevated PGE_2_ levels are closely associated with various diseases including osteoarthritis, tumors, atherosclerosis, inflammatory bowel disease, and autoimmune disorders. In ankle osteoarthritis, aberrant release of PGE_2_ disrupts the bone-brain signal balance, driving disordered subchondral bone remodeling and enhancing pain perception [14,15]. In tumors, high levels of intratumoral PGE_2_ suppress antitumor immunity and contribute to tumor progression [16]. In cardiovascular diseases, abnormal increases in PGE_2_ are strongly associated with the exacerbation of hypertension, atherosclerosis, and heart disease [17,18]. Besides above common diseases, elevated PGE_2_ levels can also lead to rare diseases. Our previous studies on primary hypertrophic osteoarthropathy (PHO) revealed that loss-of-function mutations in SLCO2A1 or HPGD genes impair PGE_2_ transport or degradation, leading to systemic PGE_2_ accumulation and manifestations such as digital clubbing, joint swelling, and accelerated bone turnover [19,20,21,22]. Targeting elevated PGE_2_ with cyclooxygenase-2 (COX-2) inhibitors is effective in PHO [21,22,23]. Given its pathological significance, PGE_2_ is a promising biomarker for monitoring disease activity and treatment response. For instance, analyzing the degree of PGE_2_ elevation can assist in assessing both the disease burden of PHO and the efficacy of COX-2 inhibitor therapy [21,22,23]. Additionally, since other commonly used non-steroidal anti-inflammatory drugs (NSAIDs) exert analgesic effects by inhibiting PGE_2_ synthesis, tracking PGE_2_ levels can help optimize their dosing and confirm efficacy.

Direct measurement of blood PGE_2_ is challenging due to its short plasma half-life [24]. Instead, PGE_2_ and its metabolite (PGE metabolite, PGEM) are rapidly excreted in urine, making 24 h urinary PGE_2_ (U-PGE_2_) and PGEM (U-PGEM) stable, non-invasive markers of systemic PGE_2_ production [25]. Most existing studies have used spot urine samples and focused on patient cohorts (e.g., cancer, renal diseases), while large-scale data on the clinical determinants of 24 h U-PGE_2_ and U-PGEM in adults are lacking [26,27]. Nevertheless, establishing normative reference data in the general population constitutes an essential prerequisite for the reliable clinical application of urinary PGE_2_ and PGEM as biomarkers. In the absence of well-characterized physiological ranges and key influencing factors, the interpretation of elevated levels in disease states remains inherently ambiguous.

Furthermore, despite the well-recognized role of PGE_2_ in bone metabolism, the relationships between 24 h U-PGE_2_, U-PGEM and key bone metabolism markers, including serum calcium, phosphorus, urinary calcium excretion, parathyroid hormone (PTH), 25-hydroxyvitamin D [25(OH)D], and bone turnover markers (BTMs), remain unexplored in the general population. Clarifying these links could establish a baseline for future skeletal disease research, and aid early detection and therapy evaluation, especially for PGE_2_-targeted interventions.

Therefore, this multicenter cross-sectional study was designed to identify the clinical associations (e.g., age, sex, BMI, lifestyle factors) of 24 h U-PGE_2_ and U-PGEM in Chinese adults, and to investigate their independent associations with calcium-phosphorus homeostasis and bone turnover markers. The ultimate aim is to establish a robust normative framework that will facilitate the future application of these urinary biomarkers in the diagnosis, monitoring, and management of skeletal and other PGE_2_-related diseases.

## 2. Materials and Methods

### 2.1. Study Population

This multi-center, cross-sectional study was registered with CHICTR.ORG.CN (ChiCTR2200056577), approved by the Ethics Committee of the Shanghai Sixth People’s Hospital (approval number: 2021-233) and conducted in accordance with the Declaration of Helsinki. Written informed consent was obtained from all participants prior to enrollment. All participants (aged > 18 years) were recruited from 9 tertiary care hospitals in China from March 2022 to March 2023. Subjects with the following conditions were excluded: (1) serious diseases affecting the pulmonary, cardiovascular, gastrointestinal, hematopoietic, renal or nervous systems; (2) conditions known to affect bone metabolism, such as osteogenesis imperfecta, Paget’s disease of bone, primary hyperparathyroidism, rheumatoid arthritis or malignant tumors; (3) concurrent use of medications known to influence urinary PGE2 levels or bone metabolism (e.g., cyclooxygenase inhibitors, diuretics, synthetic steroid hormones, epinephrine or anticonvulsants); or (4) pregnancy or lactation.

### 2.2. Clinical Data and Sample Collections

Detailed clinical information of all participants was collected and documented, including demographic characteristics (gender, age, ethnicity), anthropometric parameters (height, weight), and medical history (past disease history, medication use history). Blood samples were collected from all participants fasted for 8 to 12 h in the morning from 7:00 a.m. to 10:00 a.m., and the separated serum aliquots were stored at −80 °C until being assayed. The 24 h urine samples were collected strictly following the instructions: the initial urination at 8:00 a.m. was discarded, marking the start of the 24 h collection period, and then all urine thereafter was collected into a 5 L clean medical container until 8:00 a.m. the next day. The collected urine was stored in a cool place or refrigerated at 2–8 °C to avoid direct sunlight, with preservative (3–5 g of benzoic acid) added if necessary. After collection, the urine sample was thoroughly mixed using a stirring bar, aliquoted into the 10 mL storage tubes with a dropper, and brought to the clinic and stored at −80 °C until being assayed. Seasonality of urine sample collection was defined as spring (March to May), summer (June to August), autumn (September to November) and winter (December to February). Urine collection was postponed if participants had fever, urinary tract infection or menstruation.

### 2.3. Laboratory Assays

All blood specimens were sent to the central laboratory for biochemical evaluation, and the laboratory is accredited under CNAS MT0048 (15189). Routine liver and renal function, including alkaline phosphatase (ALP), alanine aminotransferase (ALT), creatinine (Cr), uric acid (UA), and urea nitrogen (BUN), electrolyte levels including serum calcium and phosphorus, and urinary calcium excretion (U-CaE) were measured by spectrophotometry and ion-selective electrode method, respectively (Roche Diagnostics, Basel, Switzerland). BTMs including β-isomerized C-terminal telopeptide of type I collagen (β-CTX-I) and total procollagen type 1 N-propeptide (P1NP), and intact parathyroid hormone (PTH), 25-hydroxyvitamin D [25(OH)D] were tested using electrochemiluminescence immunoassay (Roche Diagnostics, Basel, Switzerland). The 24 h U-PGE_2_ and U-PGEM levels were detected using competitive enzyme-linked immunosorbent assays (ELISA; Cayman Chemicals, Ann Arbor, MI, USA; item 500141 for PGE_2_ and item 514531 for PGEM) according to the manufacturer’s instructions as described in our previous study (19). Prior to analysis, urine samples were centrifuged at 4 °C for 10 min, and the supernatants were then extracted and diluted to three different concentrations using the diluent provided in the ELISA kit. The intra-assay coefficients of variation (CVs) for U-PGE_2_ and U-PGEM were 4.6% and 5.9%, respectively, while the inter-assay CVs for U-PGE_2_ and U-PGEM were 4.7% and 8.3%, respectively. To account for variations in urine concentration, U-PGE_2_ and U-PGEM levels were normalized to 24 h urinary creatinine (U-Cr) concentrations, which were quantified using the creatinine oxidase method (Roche Diagnostics, Basel, Switzerland).

### 2.4. Dietary Intake

Dietary calcium intake was assessed using a 1-week food frequency questionnaire [28]. Participants were required to complete a comprehensive dietary questionnaire, detailing their food intake, portion sizes, and frequency of consumption on both a daily and weekly basis. To minimize significant fluctuations in 24 h U-CaE due to dietary changes, participants were instructed to maintain detailed dietary logs on the day of baseline urine collection and adhere to their baseline diet as closely as possible during subsequent urine collection periods. The daily calcium intake per person was calculated with reference to the Chinese Food Composition Table [28].

### 2.5. Statistical Analysis

Normality was tested using the Kolmogorov–Smirnov test. Normally distributed variables were expressed as the mean ± SD, while the skewed distributed variables were expressed as median (25th and 75th percentiles). Violin plots were generated to visualize the distribution of 24 h U-PGE_2_ and U-PGEM across different age groups. The Kruskal–Wallis H test was employed to compare these distributions, followed by post hoc pairwise comparisons using Bonferroni correction to account for multiple testing. Given their skewed distributions, 24 h U-PGE_2_ and U-PGEM concentrations were log10-transformed prior to regression analyses. Multivariable linear regression was used to reveal the relationships between covariates and 24 h U-PGE_2_ and U-PGEM. The analysis was conducted using an unadjusted model and a model that adjusted for age, sex, BMI, smoking, alcohol consumption, diet calcium, diabetes, hypertension, season, calcium and vitamin D supplementation. As a sensitivity analysis, we applied restricted cubic splines (RCS) to analysis the association between age and absolute 24 h U-PGE_2_ and U-PGEM excretion, calculated as urinary analyte concentration multiplied by the corresponding 24 h urine volume. The dose–response relationship of serum calcium, serum phosphorus, U-CaE, β-CTX-I, P1NP, 25(OH)D, PTH with 24 h U-PGE_2_ and U-PGEM were analyzed using RCS fitting multiple linear regression models. RCS models were fitted using three knots placed at the 10th, 50th and 90th percentiles of each continuous exposure variable. Overall association and non-linearity were evaluated using joint tests of the spline terms. To account for multiplicity in the RCS analyses, *p*-values were adjusted using the Benjamini–Hochberg false discovery rate procedure. Additional subgroup analyses were performed after stratification by sex and age group. Among women, analyses were further stratified according to menopausal status. All analyses were conducted using R version 4.3.0 and IBM SPSS Statistics version 26.0, with a two-tailed *p*-value < 0.05 considered statistically significant.

## 3. Results

### 3.1. General Characteristics of the Study Population

**Figure 1:**
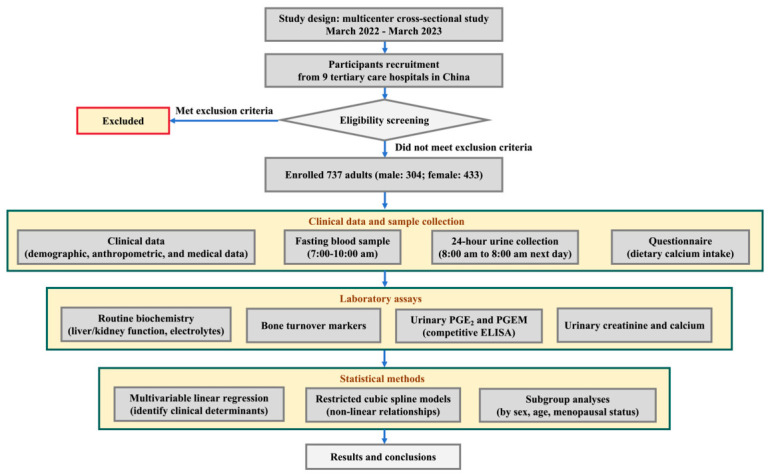
Flowchart of the study process.

**Figure 2:**
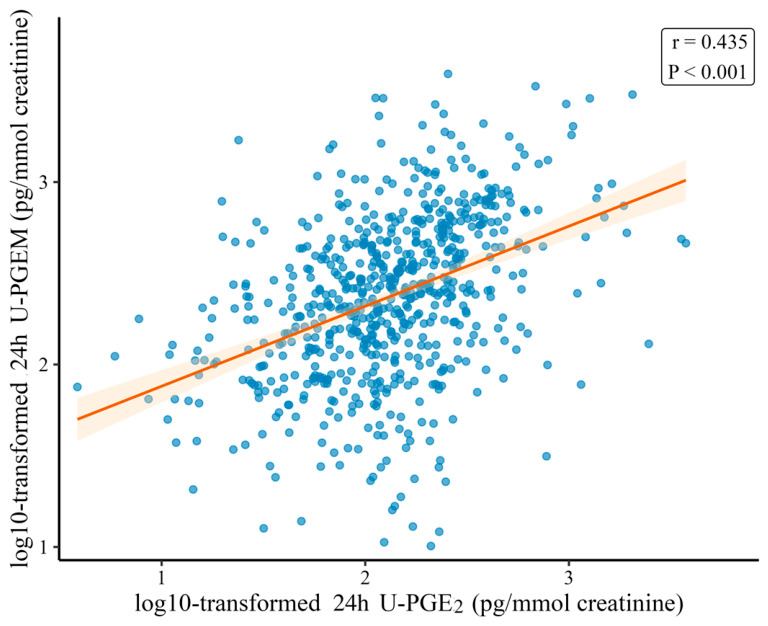
Correlation between 24 h urinary PGE_2_ and PGEM in the total population.

A comprehensive flowchart illustrating the study design, participant recruitment, sample collection, and analytical procedures is shown in Figure 1. The baseline characteristics of the total 737 participants are summarized in Table 1. All the participants (men: 304; women: 433) had a mean age of 48.0 years and a mean BMI of 23.29 kg/m^2^. The median 24 h U-PGE_2_ and U-PGEM in the total population were 133.87 and 246.76 pg/mmol creatinine, respectively, and they were moderately correlated (r = 0.43, *p* < 0.001) (Figure 2). Specifically, the median 24 h U-PGE_2_ was 133.62 pg/mmol creatinine in men and 134.33 pg/mmol creatinine in women, while the median 24 h U-PGEM was 249.00 pg/mmol creatinine in men and 242.01 pg/mmol creatinine in women. There were no significant sex differences in 24 h U-PGE_2_ (*p* = 0.511) or U-PGEM (*p* = 0.528).

### 3.2. Age-Dependent Trends in 24-h U-PGE_2_ and U-PGEM Excretion

**Figure 3:**
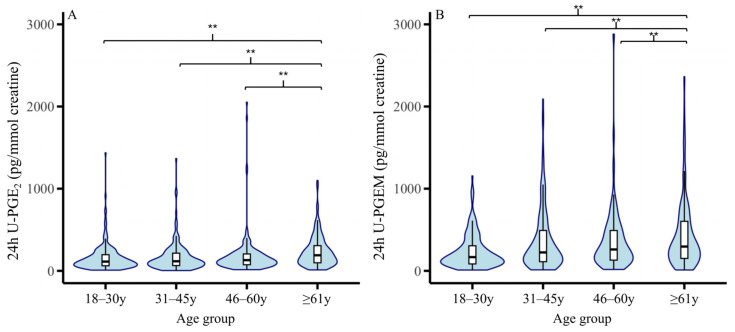
Distribution of 24 h U-PGE_2_ and U-PGEM by age group. (**A**) 24 h U-PGE_2_; (**B**) 24 h U-PGEM. The numbers of participants in the 18–30, 31–45, 46–60, and ≥61 years groups were 187, 156, 174, and 220, respectively. Overall differences across age groups were assessed using the Kruskal–Wallis test, followed by Bonferroni-adjusted post hoc pairwise comparisons. ** *p* < 0.01. U-PGE_2_, urinary prostaglandin E_2_; U-PGEM, urinary prostaglandin E metabolite.

Both biomarkers exhibited a significant age-dependent increase (Figure 3). Specifically, median 24 h U-PGE_2_ levels rose from 113.88 pg/mmol creatinine in the 18–30 age group to 191.28 pg/mmol creatinine in the ≥61 age group (*p* < 0.01). A more pronounced age-related increase was observed for 24 h U-PGEM, with median values increasing from 167.60 pg/mmol creatinine to 299.47 pg/mmol creatinine across the same age strata (*p* < 0.01). In sensitivity analyses, age remained positively associated with both 24 h PGE_2_ and PGEM excretion (Overall *p* = 0.001 and *p* < 0.001, respectively; Supplementary Figure S1A,B).

### 3.3. Factors Associated with 24-h U-PGE_2_ and U-PGEM

Multivariable linear regression analyses were performed to identify factors associated with 24 h U-PGE_2_ and U-PGEM excretion. The models included clinical and demographic covariates, lifestyle factors, comorbidities, season, dietary and supplementary intakes, as well as biochemical markers. The results of multivariable regression analysis are presented in Table 2. in Model 2, we observed that 24 h U-PGE_2_ was positively associated with age (β = 0.005, *p* < 0.001) but negatively associated with BMI (β = −0.008, *p* = 0.020), serum ALT (β = −0.002, *p* = 0.041) and serum calcium (β = −0.129, *p* = 0.006). Furthermore, similar results were observed for 24 h U-PGEM. U-PGEM remained positively associated with age (β = 0.005, *p* < 0.001) and negatively associated with serum calcium (β = −0.144, *p* = 0.008) in Model 2. However, BMI and ALT were not significantly associated with U-PGEM in the adjusted model. Using spring as the reference season, summer was associated with higher 24 h U-PGE_2_ (β = 0.206, *p* < 0.001) and U-PGEM (β = 0.136, *p* = 0.001) levels, whereas autumn remained associated only with higher U-PGE_2_ (β = 0.210, *p* < 0.001) after multivariable adjustment.

**Figure 4:**
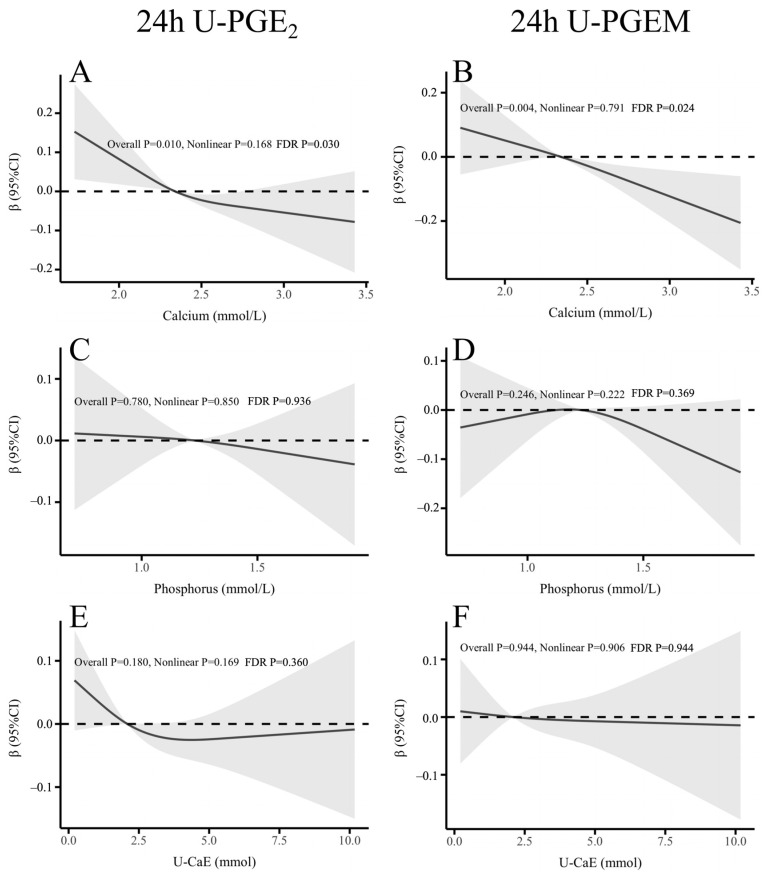
Relationship of 24 h U-PGE_2_ and U-PGEM with serum calcium, phosphorus and U-CaE. Restricted cubic splines were utilized to flexibly model the association between 24 h U-PGE_2_ and (**A**) serum calcium, (**C**) phosphorus, (**E**) U-CaE, and the association between 24 h U-PGEM and (**B**) serum calcium, (**D**) phosphorus, (**F**) U-CaE, adjusted for age, sex, BMI, smoking, alcohol consumption, diet calcium, diabetes, hypertension, season, calcium and vitamin D supplementation. U-PGE_2_, urinary prostaglandin E_2_; U-PGEM, urinary prostaglandin E metabolite; U-CaE, urinary calcium excretion.

To explore potential dose–response relationships, RCS were employed to model the associations of serum calcium, phosphorus, U-CaE levels with 24 h U-PGE_2_ and U-PGEM (Figure 4). The results revealed a significant inverse linear association between serum calcium levels and both 24 h U-PGE_2_ (Overall *p* = 0.010, FDR-adjusted *p* = 0.030; Figure 4A) and U-PGEM (Overall *p* = 0.004, FDR-adjusted *p* = 0.024; Figure 4B). Furthermore, the tests for non-linearity were not statistically significant (Nonlinear *p* = 0.168 and *p* = 0.791, respectively), confirming that these dose–response relationships are predominantly linear. However, neither 24 h U-PGE_2_ nor U-PGEM exhibited any statistically significant linear or non-linear associations with serum phosphorus or U-CaE levels (all Overall *p* > 0.05; Figure 4C–F).

### 3.4. Relationships of 24-h U-PGE_2_ and U-PGEM with Bone Metabolism Markers

**Figure 5:**
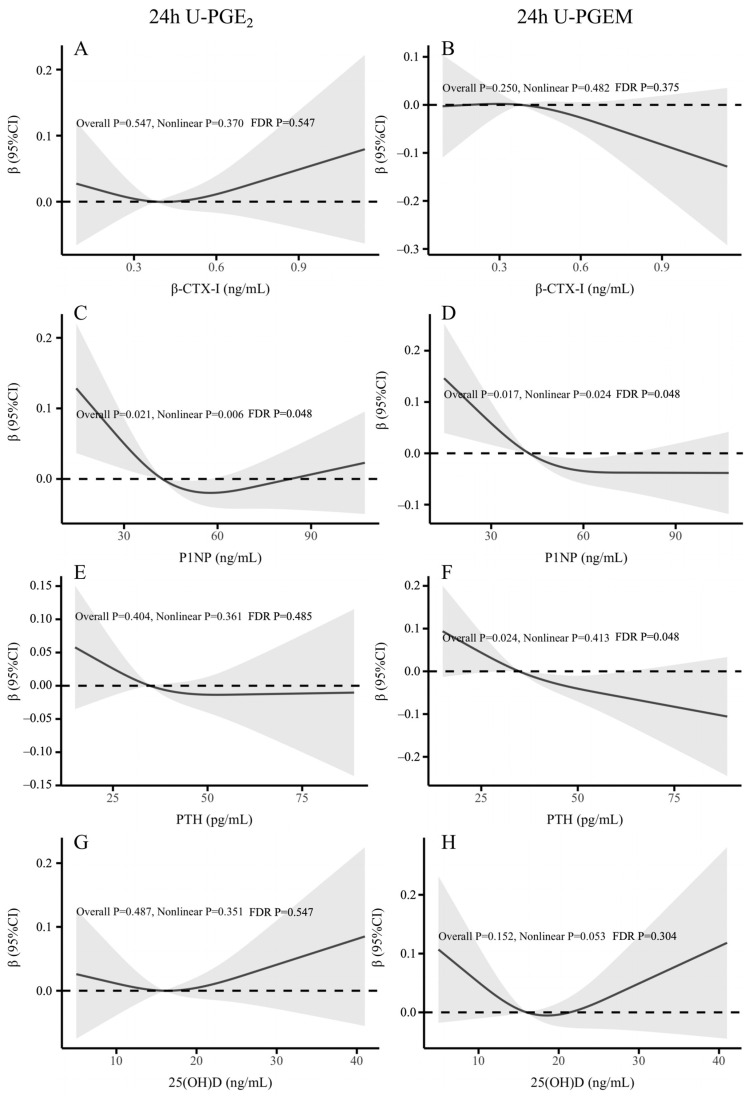
Relationship of 24 h U-PGE_2_ and U-PGEM with β-CTX-I, P1NP, 25(OH)D and PTH. Restricted cubic splines were utilized to flexibly model the association between 24 h U-PGE_2_ and (**A**) β-CTX-I, (**C**) P1NP, (**E**) PTH and (**G**) 25(OH)D, and the association between 24 h U-PGEM and (**B**) β-CTX-I, (**D**) P1NP, (**F**) PTH and (**H**) 25(OH)D. U-PGE_2_, urinary prostaglandin E_2_; U-PGEM, urinary prostaglandin E metabolite; β-CTX-I, β-isomerized C-terminal telopeptide of type I collagen; P1NP, total procollagen type 1 N-propeptide; PTH, intact parathyroid hormone; 25(OH)D, 25-hydroxyvitamin D.

To further investigate the interplay between systemic PGE_2_ production and bone metabolism, RCS were utilized to model the associations of 24 h U-PGE_2_ and U-PGEM with bone turnover markers (Figure 5).

The analyses revealed a significant non-linear association between P1NP and both 24 h U-PGE_2_ (Overall *p* = 0.021, Nonlinear *p* = 0.006, FDR-adjusted *p* = 0.048; Figure 5C) and U-PGEM (Overall *p* = 0.017, Nonlinear *p* = 0.024, FDR-adjusted *p* = 0.048; Figure 5D). The fitted curves showed lower estimated U-PGE_2_ and U-PGEM levels across increasing P1NP concentrations in the lower-to-middle range, with a flatter pattern at higher P1NP concentrations.

24 h U-PGEM demonstrated a significant inverse linear association with PTH levels (Overall *p* = 0.024, Nonlinear *p* = 0.413, FDR-adjusted *p* = 0.048; Figure 5F), whereas the association for U-PGE_2_ was not statistically significant (Overall *p* = 0.404, FDR-adjusted *p* = 0.485; Figure 5E). Furthermore, neither 24 h U-PGE_2_ nor U-PGEM exhibited any statistically significant linear or non-linear associations with β-CTX-I (Figure 5A,B) or 25(OH)D levels (all FDR-adjusted *p* > 0.05; Figure 5G,H).

In sex-stratified and age-stratified analyses, the overall direction of the associations between 24 h U-PGE_2_/U-PGEM and the main bone metabolism markers was broadly consistent with that observed in the total population (Supplementary Figures S2–S5). Among women, similar patterns were observed in premenopausal and postmenopausal subgroups (Supplementary Figure S6).

## 4. Discussion

In this multicenter study of 737 Chinese adults, 24 h urinary PGE_2_ and PGEM increased with age and in summer/autumn, and were inversely linearly associated with serum calcium. BMI and ALT were negatively associated only with U-PGE_2_. Both biomarkers showed a non-linear threshold relationship with P1NP (steep decline then plateau). U-PGEM, but not U-PGE_2_, was inversely associated with intact PTH. No associations were found with serum phosphorus, U-CaE, β-CTX-I, or 25(OH)D. These findings offer a normative baseline essential for interpreting PGE_2_ status, and reveal physiological links between systemic PGE_2_ production and bone metabolism.

In this study, median 24 h U-PGE_2_ and U-PGEM were 133.87 and 246.76 pg/mmol creatinine, respectively, with no sex difference. Existing data on urinary PGE_2_ and PGEM are limited, mostly from spot urine or patient cohorts [21,25,29]. Given the clear circadian variation with a 30% decrease at night, 24 h urine better reflects daily average than spot samples [30]. The pronounced, positive associations between age and both 24 h U-PGE_2_ and U-PGEM were revealed, which aligns with the concept of “inflammaging”, a state of chronic, low-grade inflammation that develops with aging. Similar findings were reported by Geurts et al. and Wen et al. in population-based cohorts [25,31]. Although circulating and excreted PGE_2_ and PGEM levels increase with age, tissue-specific levels exhibit considerable heterogeneity. In aging mice, elevated COX-2 expression and PGE_2_ production were higher in macrophages, lung tissue, and kidneys, and activation of the PGE_2_-EP2 axis in myeloid cells was shown to impair cognition [9,32,33]. Conversely, in aged muscle and cartilage, elevated 15-hydroxyprostaglandin dehydrogenase (15-PGDH) reduced PGE_2_ signaling, and its inhibition rejuvenated muscle mass and promoted cartilage regeneration [34,35]. These divergent observations underscore the complex, context-dependent nature of PGE_2_ signaling in the aging process. Interestingly, we also found higher excretion levels in summer and autumn. Seasonal variations in PGE_2_ have been previously reported in the context of allergic rhinitis, but our observation in Chinese adults suggests that environmental factors such as temperature, sunlight exposure, or dietary changes may influence systemic PGE_2_ production [36]. Although seasonal differences in urinary PGE_2_ and PGEM were noted, the unbalanced sampling, especially few winter collections, limits distinguishing true seasonality from confounding. These exploratory findings thus require validation in studies with balanced seasonal recruitment.

Our study identified a significant negative correlation between urinary PGE_2_ and PGEM excretion and serum calcium, but no association with urinary calcium excretion. This dissociation may reflect the interplay of direct physiological actions and homeostatic compensation. On one hand, elevated PGE_2_ can promote renal calcium excretion by inhibiting the Na^+^-K^+^-2Cl^−^ cotransporter and reduce intestinal calcium absorption by suppressing 1α-hydroxylase activity [37,38]. On the other hand, lower serum calcium may stimulate PGE^2^ production as a compensatory mechanism to mobilize calcium stores [39]. These bidirectional pathways could collectively underlie the observed negative correlation. The absence of an association with urinary calcium might be explained by the kidney’s powerful homeostatic capacity. In healthy individuals, other compensatory mechanisms, such as PTH regulating tubular reabsorption, may buffer any direct renal effect of PGE_2_. Moreover, within the narrow physiological range, the impact of PGE_2_ on urinary calcium is likely overshadowed by dominant confounding factors such as dietary calcium and sodium intake.

Regarding bone metabolism, PGE_2_ is known to exert dual effects in promoting both bone formation and bone loss to maintain balance. However, the exact relationships between 24 h urinary PGE_2_ and PGEM excretion with bone metabolism markers were not clear. In this study, a novel threshold-type non-linear association was observed between P1NP and both U-PGE_2_ and U-PGEM. At low P1NP levels, increasing bone formation was accompanied by a steep decline in PGE_2_ excretion, while at high P1NP levels, the relationship plateaued. Although the fitted curves suggested an inverse pattern that became less pronounced at higher P1NP concentrations, the cross-sectional design precludes conclusions regarding temporality or causality. These findings may reflect bidirectional biological relationships, reverse causation, residual confounding, or other unmeasured mechanisms. Therefore, the observed pattern should be considered hypothesis-generating rather than evidence of a compensatory PGE_2_ response to low bone formation [3,40,41]. U-PGEM, but not U-PGE_2_, was inversely and linearly associated with PTH. Although PTH stimulates PGE_2_ production in bone and kidney, the inverse association may be explained by a negative feedback loop that PTH raises serum calcium, which in turn suppresses PGE_2_ synthesis [42,43]. The lack of significance for U-PGE_2_ may reflect better systemic representation of U-PGEM. Given U-PGE_2_ ELISA’s susceptibility to degradation and cross-reactivity, unlike the more stable PGEM, the U-PGE_2_ null results should be interpreted cautiously due to potential measurement error. Neither biomarker was associated with β-CTX-I, 25(OH)D, or urinary calcium excretion, indicating that systemic PGE_2_ production is more closely linked to bone formation and its regulators than to resorption or vitamin D status. The high prevalence of vitamin D insufficiency in our cohort may also influence bone turnover markers and PGE_2_/PGEM associations, so residual confounding related to vitamin D status cannot be completely ruled out.

Several limitations should be acknowledged. The cross-sectional design precludes causal inference, and the modest sample size, together with the exclusively Chinese cohort from tertiary care hospitals limits statistical precision and generalizability. We did not assess physical activity, hormone replacement therapy (HRT) use or other inflammatory markers such as other prostaglandin metabolites and cytokines, which could have influenced the PGE_2_-bone metabolism associations or provided a broader inflammatory context. Regarding calcium-related measures, serum albumin and ionized calcium were unavailable, preventing albumin-adjusted calcium calculations; additionally, dietary calcium was estimated via a 1-week self-reported FFQ, which is subject to recall error and potential underestimation, and may have attenuated the adjusted associations. Although the subgroup analyses showed broadly consistent patterns, other unmeasured lifestyle factors cannot be excluded. Thus, the lack of independent associations in adjusted models should not be interpreted as evidence against biological relevance. Future community-based representative studies with comprehensive assessment of reproductive status, sex hormones, lifestyle, and inflammatory biomarkers are warranted.

## 5. Conclusions

In this multicenter study of healthy adults, age, season, and serum calcium were independent determinants of 24 h U-PGE_2_ and U-PGEM. Both biomarkers showed a non-linear threshold relationship with P1NP and an inverse linear relationship with serum calcium, and U-PGEM was also inversely associated with PTH. These findings highlight complex interactions between systemic PGE_2_ production, calcium homeostasis, and bone formation in healthy individuals.

## Figures and Tables

**Table 1:** Basic characteristics of the 737 participants.

Characteristics	Total Sample (*n* = 737)	Men (*n* = 304)	Women (*n* = 433)	*p*
Age (years)	48.00 ± 17.80	48.90 ± 17.65	47.38 ± 17.90	0.168
Height (cm)	163.98 ± 8.36	170.38 ± 7.02	159.56 ± 6.03	**<0.001**
Weight (kg)	62.79 ± 13.06	70.00 ± 12.78	57.80 ± 10.72	**<0.001**
BMI (kg/m^2^)	23.29 ± 4.23	24.09 ± 4.20	22.73 ± 4.16	**<0.001**
Current smokers (*n*, %)	68 (9.2)	62 (20.4)	6 (1.4)	**<0.001**
Alcohol consumption (*n*, %)	65 (8.8)	49 (16.1)	16 (3.7)	**<0.001**
Diabetes (*n*, %)	43 (5.8)	21 (6.9)	22 (5.1)	0.378
Hypertension (*n*, %)	118 (16.0)	48 (15.8)	70 (16.2)	0.972
Diet calcium (mg)	232.73 (77.50, 485.89)	231.60 (65.10, 487.90)	236.60 (81.32, 485.89)	0.999
Calcium supplementation (*n*, %)	97 (13.2)	37 (12.2)	60 (13.9)	0.578
Vitamin D supplementation (*n*, %)	74 (10.0)	30 (9.9)	44 (10.2)	0.995
Season (*n*, %)				0.071
Spring	176 (23.9)	78 (25.7)	98 (22.6)	
Summer	382 (51.8)	141 (46.4)	241 (55.7)	
Autumn	173 (23.5)	83 (27.3)	90 (20.8)	
Winter	6 (0.8)	2 (0.7)	4 (0.9)	
ALP (U/L)	71.0 (55.0, 86.5)	74.5 (61.0, 89.0)	66.0 (53.0, 83.0)	**<0.001**
ALT (U/L)	12.0 (9.0, 19.0)	16.0 (10.0, 25.0)	11.0 (8.0, 15.0)	**<0.001**
BUN (mmol/L)	4.6 (3.8, 5.5)	4.9 (4.1, 5.8)	4.4 (3.7, 5.2)	**<0.001**
UA (μmol/L)	295.0 (242.0, 367.0)	355.0 (291.0, 418.0)	271.0 (224.0, 316.5)	**<0.001**
Cr (μmol/L)	66.0 (58.0, 80.0)	80.0 (67.0, 91.0)	60.0 (54.0, 68.0)	**<0.001**
Calcium (mmol/L)	2.34 (2.23, 2.46)	2.33 (2.24, 2.45)	2.35 (2.23, 2.46)	0.603
Phosphorus (mmol/L)	1.23 (1.07, 1.38)	1.15 (1.01, 1.33)	1.28 (1.13, 1.40)	**<0.001**
U-CaE (mmol)	2.09 (1.20, 3.42)	2.26 (1.26, 4.18)	1.98 (1.19, 3.14)	**0.017**
β-CTX-I (ng/mL)	0.38 (0.26, 0.56)	0.41 (0.28, 0.58)	0.36 (0.25, 0.53)	**0.009**
P1NP (ng/mL)	42.6 (32.0, 56.5)	42.4 (31.7, 56.6)	42.6 (33.1, 55.8)	0.655
25(OH)D (ng/mL)	16.0 (12.0, 21.0)	18.0 (13.0, 24.0)	14.0 (11.0, 19.0)	**<0.001**
PTH (pg/mL)	34.95 (26.90, 44.40)	35.5 (26.9, 45.3)	34.5 (27.0, 44.1)	0.608
U-PGE_2_ (pg/mmol creatinine)	133.87 (73.75, 239.23)	133.62 (75.34, 253.65)	134.33 (70.61, 230.72)	0.511
U-PGEM (pg/mmol creatinine)	246.76 (119.32, 475.44)	249.00 (115.28, 475.07)	242.01 (120.77, 480.32)	0.528

BMI, body mass index; ALP, alkaline phosphatase; ALT, alanine aminotransferase; BUN, urea nitrogen; UA, uric acid; Cr, creatinine; U-CaE, urinary calcium excretion; β-CTX-I, β-isomerized C-terminal telopeptide of type I collagen; P1NP, total procollagen type 1 N-propeptide; 25(OH)D, 25-hydroxyvitamin D; PTH, intact parathyroid hormone; U-PGE_2_, urinary prostaglandin E_2_; U-PGEM, urinary prostaglandin E metabolite. Significant values (*p* < 0.05) are presented in bold.

**Table 2:** Factors associated with 24 h U-PGE_2_ and U-PGEM.

	24 h U-PGE_2_				24 h U-PGEM			
	Model 1		Model 2		Model 1		Model 2	
	B (95% CI)	*p*	β (95% CI)	*p*	β (95% CI)	*p*	β (95% CI)	*p*
Sex (Women)	−0.034 (−0.095, 0.027)	0.279	−0.045 (−0.107, 0.017)	0.155	−0.019 (−0.089, 0.050)	0.580	−0.016 (−0.089, 0.057)	0.664
Age (years)	0.005 (0.003, 0.006)	**<0.001**	0.005 (0.003, 0.007)	**<0.001**	0.005 (0.003, 0.007)	**<0.001**	0.005 (0.003, 0.007)	**<0.001**
BMI (kg/m^2^)	−0.005 (−0.012, 0.002)	0.149	−0.008 (−0.016, −0.001)	**0.020**	0.001 (−0.007, 0.009)	0.844	−0.003 (−0.011, 0.005)	0.464
Smoking	−0.003 (−0.107, 0.100)	0.949	−0.017 (−0.131, 0.096)	0.765	0.064 (−0.052, 0.180)	0.276	0.042 (−0.090, 0.173)	0.535
Drinking	−0.029 (−0.135, 0.076)	0.585	−0.011 (−0.123, 0.100)	0.842	−0.014 (−0.133, 0.104)	0.812	−0.024 (−0.153, 0.105)	0.715
Diet calcium (per 100 mg)	−0.013 (−0.020, −0.005)	**0.002**	−0.005 (−0.013, 0.002)	0.162	−0.008 (−0.017, 0.001)	0.062	−0.002 (−0.011, 0.007)	0.614
Diabetes	0.070 (−0.059, 0.198)	0.287	−0.052 (−0.187, 0.083)	0.452	0.108 (−0.033, 0.249)	0.132	−0.052 (−0.207, 0.103)	0.512
Hypertension	0.152 (0.071, 0.233)	**<0.001**	0.082 (−0.014, 0.177)	0.094	0.187 (0.095, 0.278)	**<0.001**	0.102 (−0.011, 0.215)	0.076
Season								
Summer	0.205 (0.132, 0.277)	**<0.001**	0.206 (0.133, 0.278)	**<0.001**	0.126 (0.042, 0.209)	**0.003**	0.136 (0.053, 0.220)	**0.001**
Autumn	0.231 (0.155, 0.316)	**<0.001**	0.210 (0.124, 0.295)	**<0.001**	0.114 (0.017, 0.212)	**0.022**	0.087 (−0.011, 0.186)	0.082
Winter	0.284 (−0.046, 0.615)	0.092	0.246 (−0.085, 0.577)	0.145	0.138 (−0.227, 0.503)	0.459	0.072 (−0.296, 0.439)	0.701
Calcium supplementation	0.076 (−0.012, 0.165)	0.091	−0.071 (−0.189, 0.047)	0.238	0.099 (−0.001, 0.199)	0.052	−0.059 (−0.197, 0.079)	0.400
Vitamin D supplementation	0.121 (0.021, 0.220)	**0.018**	0.075 (−0.059, 0.208)	0.274	0.144 (0.033, 0.255)	**0.011**	0.065 (−0.089, 0.219)	0.411
ALP (U/L)	0.000 (−0.001, 0.001)	0.444	−0.001 (−0.002, 0.001)	0.346	0.000 (−0.001, 0.002)	0.562	−0.001 (−0.002, 0.001)	0.232
ALT (U/L)	−0.001 (−0.003, 0.001)	0.306	−0.002 (−0.004, 0.001)	**0.041**	0.001 (−0.002, 0.003)	0.550	0.001 (−0.002, 0.002)	0.983
BUN (mmol/L)	0.005 (−0.011, 0.021)	0.520	−0.012 (−0.029, 0.005)	0.159	0.002 (−0.016, 0.02)	0.841	−0.022 (−0.042, −0.002)	**0.034**
UA (μmol/L)	0.001 (0.001, 0.001)	0.642	0.001 (−0.001, 0.001)	0.392	0.001 (0.001, 0.001)	0.696	0.001 (−0.001, 0.001)	0.210
Calcium (mmol/L)	−0.154 (−0.246, −0.063)	**0.001**	−0.129 (−0.220, −0.038)	**0.006**	−0.220 (−0.323, −0.117)	**<0.001**	−0.144 (−0.251, −0.038)	**0.008**
Phosphorus (mmol/L)	−0.104 (−0.223, 0.015)	0.087	0.013 (−0.105, 0.130)	0.834	−0.145 (−0.280, −0.010)	**0.036**	−0.049 (−0.187, 0.090)	0.490
U-CaE (mmol)	−0.014 (−0.028, −0.001)	**0.039**	−0.011 (−0.024, 0.003)	0.117	−0.008 (−0.023, 0.007)	0.304	−0.003 (−0.019, 0.012)	0.669

Results are based on multivariable linear regression. Model 1 is unadjusted. Model 2 is adjusted for age, sex, BMI, smoking, alcohol consumption, diet calcium, diabetes, hypertension, season, calcium and vitamin D supplementation. Season was entered into the multivariable regression models as a four-level categorical variable (spring, summer, autumn, and winter), with spring as the reference category. Significant values (*p* < 0.05) are presented in bold. U-PGE_2_, urinary prostaglandin E_2_; U-PGEM, urinary prostaglandin E metabolite; BMI, body mass index; ALP, alkaline phosphatase; ALT, alanine aminotransferase; BUN, urea nitrogen; UA, uric acid; U-CaE, urinary calcium excretion.

## Data Availability

The raw data supporting the conclusions of this article will be made available by the authors, without undue reservation, to any qualified researcher.

## Supplementary Materials

The following supporting information can be downloaded at: https://www.mdpi.com/article/10.3390/biomedicines14071547/s1, Supplementary Figure S1. Relationship of 24-h U-PGE_2_ and U-PGEM with age. Supplementary Figure S2. Relationship of 24-h U-PGE_2_ and U-PGEM with β-CTX-I, P1NP, 25(OH)D and PTH in men. Supplementary Figure S3. Relationship of 24-h U-PGE_2_ and U-PGEM with β-CTX-I, P1NP, 25(OH)D and PTH in women. Supplementary Figure S4. Relationship of 24-h U-PGE_2_ and U-PGEM with β-CTX-I, P1NP, 25(OH)D and PTH in age more than 50. Supplementary Figure S5. Relationship of 24-h U-PGE_2_ and U-PGEM with β-CTX-I, P1NP, 25(OH)D and PTH in age less than 50. Supplementary Figure S6. Relationship of 24-h U-PGE_2_ and U-PGEM with β-CTX-I, P1NP, 25(OH)D and PTH in postmenopausal women.

## Abbreviations

The following abbreviations are used in this manuscript:

PGE_2_	Prostaglandin E_2_
PHO	Primary hypertrophic osteoarthropathy
COX-2	Cyclooxygenase-2
NSAIDs	Non-steroidal anti-inflammatory drugs
PGEM	PGE metabolite
U-PGE_2_	24 h urinary PGE_2_
U-PGEM	24 h urinary PGEM
PTH	Parathyroid hormone
25(OH)D	25-hydroxyvitamin D
BTMs	Bone turnover markers
BMI	Body mass index
ALP	Alkaline phosphatase
ALT	Alanine aminotransferase
Cr	Creatinine
UA	Uric acid
BUN	Urea nitrogen
U-CaE	urinary calcium excretion
β-CTX-I	β-isomerized C-terminal telopeptide of type I collagen
P1NP	Total procollagen type 1 N-propeptide
ELISA	Enzyme-linked immunosorbent assays
CVs	Coefficients of variation
U-Cr	24 h urinary creatinine
RCS	Restrictive cubic splines
15-PGDH	15-hydroxyprostaglandin dehydrogenase
