# Supplementary material for: Clinical Determinants and Bone Metabolic Correlates of 24-h Urinary PGE2 and PGEM Excretion in Chinese Adults: A Multicenter Cross-Sectional Study
Source: Biomedicines. 2026 Jul 10;14(7):1547. doi: 10.3390/biomedicines14071547 (PMC13404794; doi:10.3390/biomedicines14071547)
Supplement: Supplementary file 1 [file biomedicines-14-01547-s001.zip › biomedicines-4337893-supplementary.pdf]

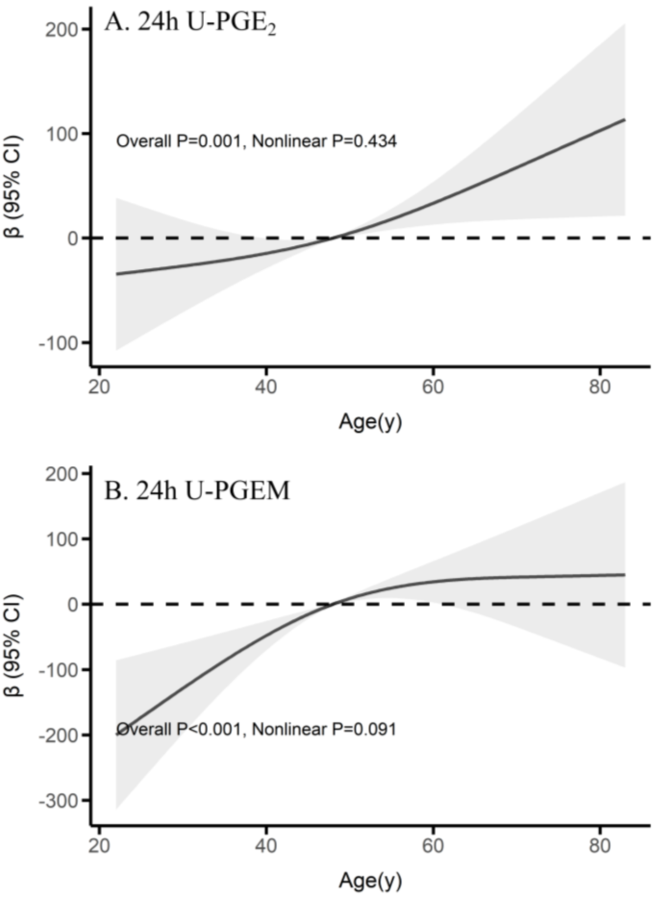

Supplementary Figure S1. Relationship of 24-hour U-PGE_2 and U-PGEM with age.

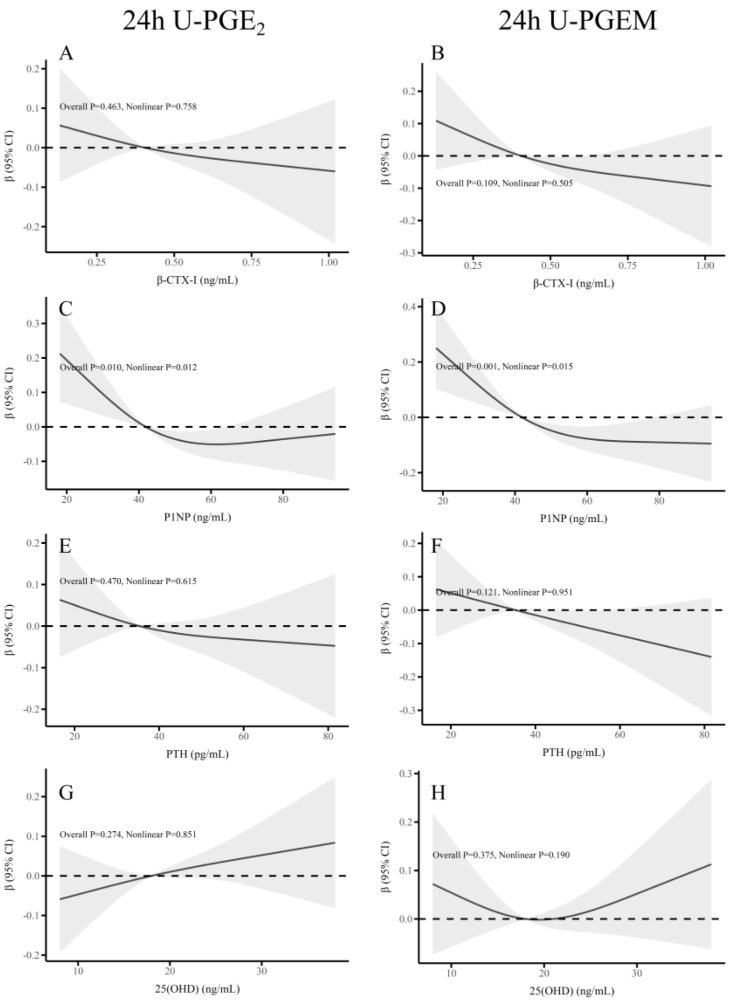

Supplementary Figure S2. Relationship of 24-hour U-PGE_2 and U-PGEM with β-CTX-I, P1NP, 25(OH)D and PTH in men.

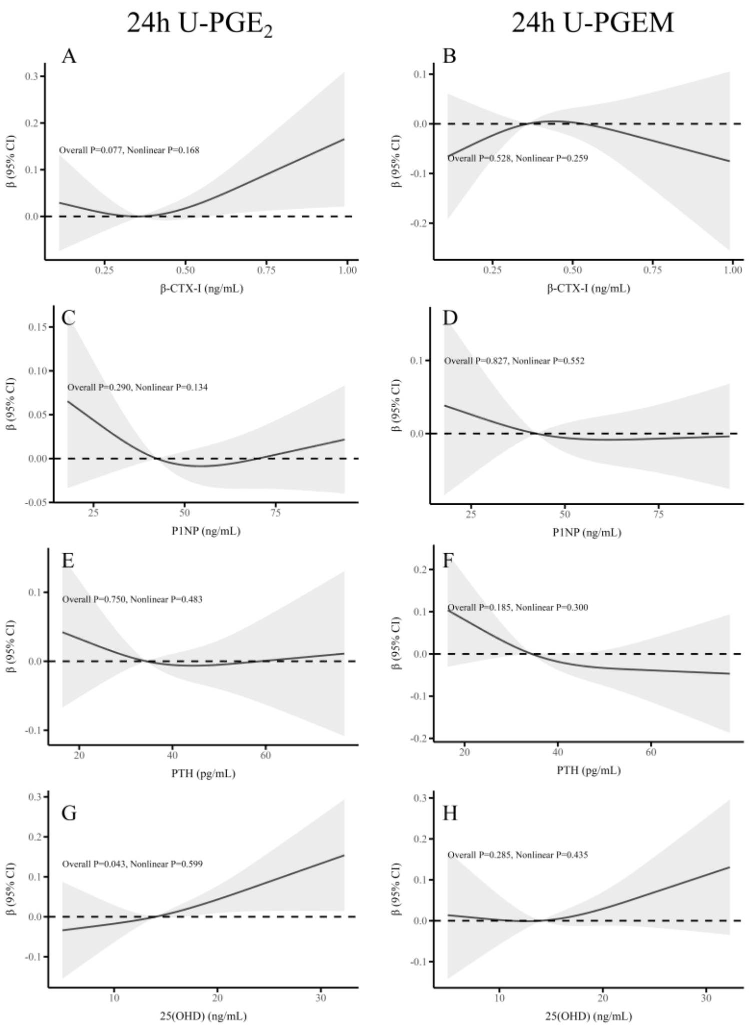

Supplementary Figure S3. Relationship of 24-hour U-PGE_2 and U-PGEM with β-CTX-I, P1NP, 25(OH)D and PTH in women.

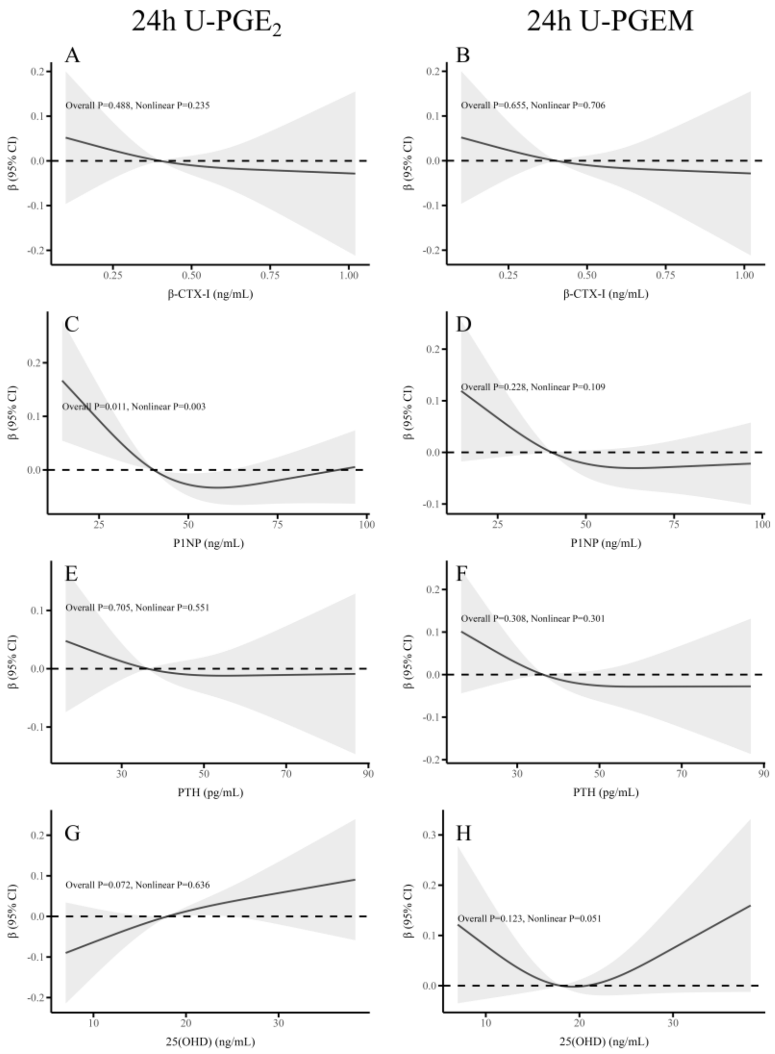

Supplementary Figure S4. Relationship of 24-hour U-PGE_2 and U-PGEM with β-CTX-I, P1NP, 25(OH)D and PTH in age more than 50.

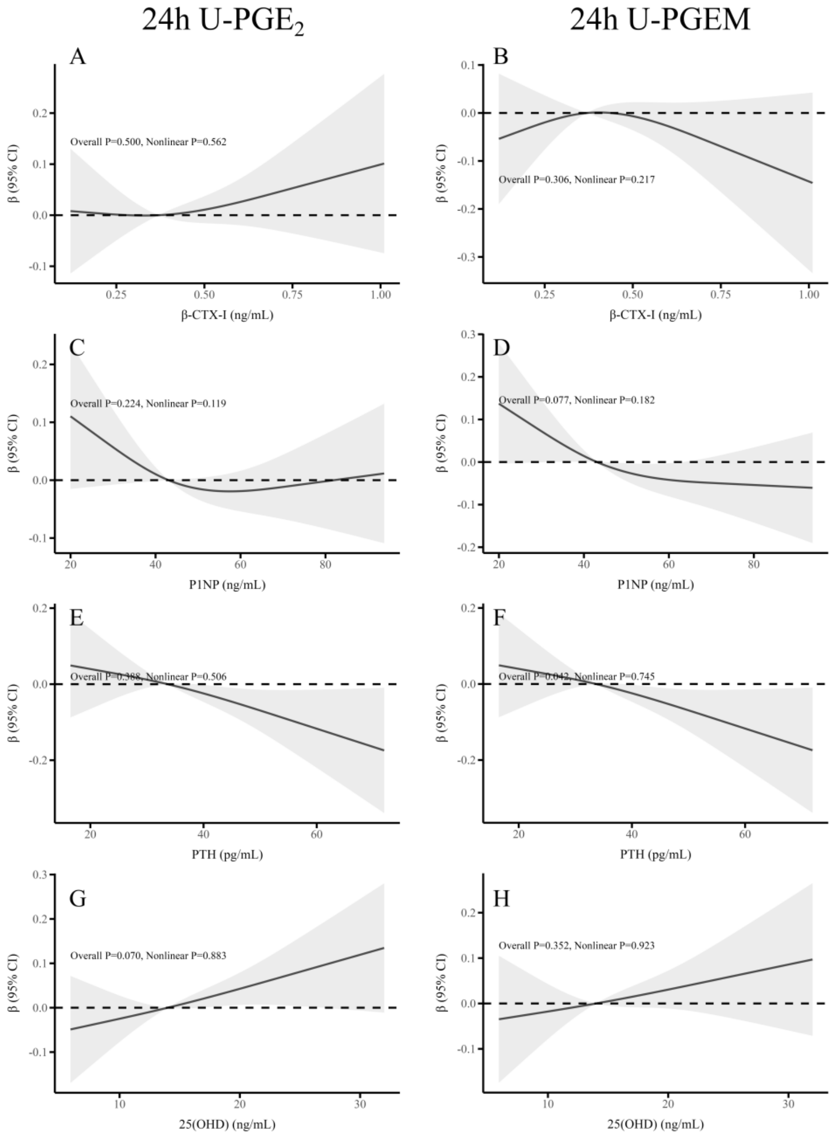

Supplementary Figure S5. Relationship of 24-hour U-PGE_2 and U-PGEM with β-CTX-I, P1NP, 25(OH)D and PTH in age less than 50.

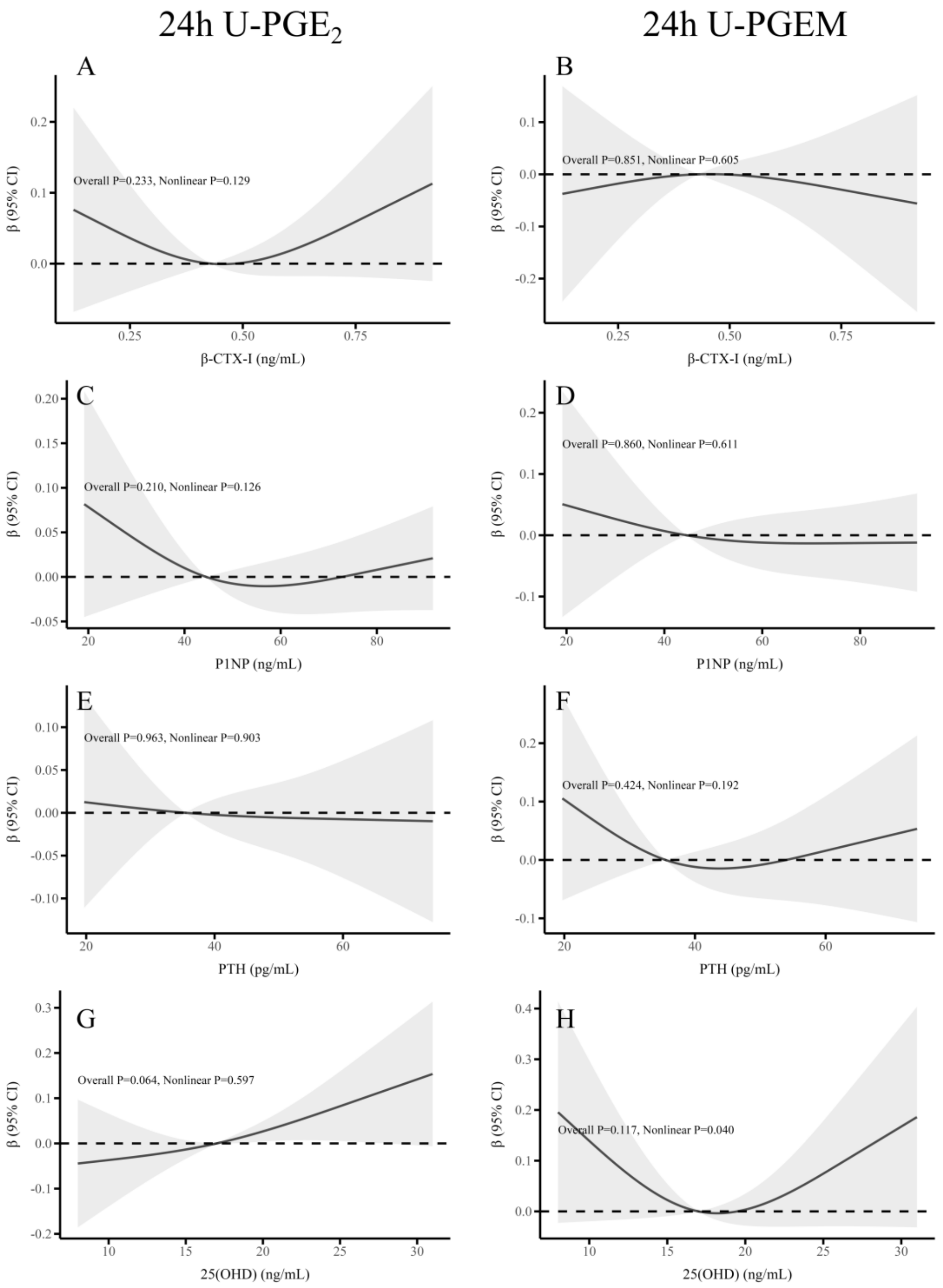

Supplementary Figure S6. Relationship of 24-hour U-PGE_2 and U-PGEM with β-CTX-I, P1NP, 25(OH)D and PTH in postmenopausal women.
